# Future Challenges in Research in Children with Neurodevelopmental Disorders

**DOI:** 10.3390/children8050328

**Published:** 2021-04-23

**Authors:** Dulce Romero-Ayuso

**Affiliations:** Department of Physical Therapy, Occupational Therapy Division, Faculty of Health Sciences, University of Granada, 18016 Granada, Spain; dulceromero@ugr.es

The prevalence of neurodevelopmental disorders exceeds 15% worldwide, and often they are associated with other neurological disorders [1]. Neurodevelopmental disorders are characterized by showing different patterns in the acquisition of motor, cognitive, linguistic and socio-emotional skills, which affect the functioning in the different contexts relevant to children. They are, therefore, a relevant topic for clinicians engaged in evaluation and intervention in children. The aims of this Special Issue are to present new approaches and results on evaluation and intervention, emphasizing the importance of the full active participation of the child in the different environments, personal, family and/or academic, that are part of their daily life. In this way, the studies included in this monograph allow us to cross the barrier of the clinical context, changing the focus of evaluation and intervention to other spaces where the main significant activities of children occur, such as play, educational learning, activities of daily life and self-care, rest and social participation. A section has also been dedicated to studies that provide more evidence on new therapies, such as the use of hypnotherapy or virtual reality systems. 

Several evaluation tools are presented in this Special Issue. The first of them is a new tool developed from Angel Riviére’s autism spectrum inventory [2] of these children, including the response to sensory stimuli [3]. According to the last diagnostic classification of DSM-5, the importance of sensorial hypersensitivity in autism spectrum disorder [4], which may result from a sensory processing disorder, has been recognized [5]. The second tool is a cross-cultural adaptation to the Spanish population of an instrument called My Child’s Play [6] that allows the evaluation of the play of children between 3 and 9 years old. This instrument, through the play, provides a cut score that helps to differentiate children with typical development and with neurodevelopmental disorders [7]. Additionally, this study enabled us to determine what factors of the play can be weaknesses or strengths: cognitive flexibility and executive attention, communication and social interaction, the preferences and characteristics of the play or the opportunities of the context. On the other hand, Sewani and Kashef [8] show an innovative proposal with a study that allows us to approach the diagnosis of autism spectrum disorders using a machine encoder based on the learning of neural networks. Finally, within the evaluation tools, a new approach is presented with the aim to develop children’s understanding of death, through the EsCoMu scale [9]. This instrument is an important contribution given the lack of research that addresses bereavement in the child. The authors provide a useful tool, with good psychometric properties and four factors underlying children’s understanding of death: universality, irreversibility, nonfunctionality and causality. 

Regarding interventions, the effects of low-intensity modified Constraint-Induced Movement Therapy on upper limb functionality in eight children with hemiplegia aged 4–8 years are presented. The results of this study show an increase in the spontaneous use of the affected upper limb in bimanual tasks and dissociated and propensity movements [10]. Likewise, the study of Riquelme, Sabater-Gárriz and Montoya [11] delves into the impact on the family of chronic pain in children with cerebral palsy (CP) and its relationship to the quality of life of these children and their ability to communicate. This volume also addresses the effectiveness of two types of emerging therapies: hypnotherapy in children with CP [12] and the use of virtual reality in children with attention-deficit hyperactivity disorder (ADHD) [13]. Both studies allow us to advance the available evidence. Thus, it is concluded that hypnotherapy, for 8–12 weeks, stimulates proprioceptive and balance reactions, reducing muscle spasticity, through rhythmic and symmetrical movement, leading to an improvement in the gross motor function in children with CP, such as lying down, roll, sitting and walking [12]. The meta-analysis of Virtual Reality-Based Interventions for Children and Adolescents with ADHD highlights the need to design randomized controlled trials with virtual reality and concludes that virtual reality may be effective in improving performance in sustained attention tasks in children with ADHD [13].

Three studies address the impact of neurodevelopmental disorders on personal, educational and social functioning. In the first of these, Blanco-Martínez et al. [14] present a cross-sectional study on participation in different everyday contexts, finding differences between children with and without neurodevelopmental disorders and their possible impact on daily life. Among the significant activities in children between the age of 6 and 12 are school activities. In this way, Maciver et al. [15] approach Scotland’s experience in developing strategies and models that promote the participation and inclusion of children with support needs at schools, through the involvement and training of teachers, in order to reduce inequalities. The proposed model, CIRCLE, incorporates the concepts of the model of human occupation, with the aim to broaden our perspective beyond deficits, including the strengths, motivation, routines of the child in a comfortable, flexible school, able to innovate, that train teachers, professionals and include families. Likewise, the concern about the care of children with autism at school is also reflected in the study of Taresh et al. [16] conducted in Yemen.

We hope the studies presented in this Special Issue will be useful and of value to the different professionals and researchers in the fields of occupational therapy, physiotherapy, psychology and education and, above all, to help improve the opportunities in the everyday contexts of children with neurodevelopmental disorders. Research along with clinical experience and clinical reasoning will enable the development of practices based on the best available evidence. Additionally, we hope that this volume serves its intended purpose: an advance in two of the challenges of the H2031 Strategy and the 2030 agenda for sustainable development, related to Goal 3 “Good health and well-being” and Goal 10 referring to “reducing inequalities”: (1) promoting the dissemination of research on factors that can provide safe, nonviolent, inclusive, and effective learning environments for all and (2) presenting interventions that reduce inequality to enhance or promote social inclusion and the improvement of the self-regulation processes of children with neurodevelopmental disorders. The challenges of the future are stimulating, and we want this Special Issue to be an initial approach to continue advancing and creating the best opportunities for the development, health and well-being of all children.
